# Relevance of ancient Indian wisdom to modern mental health – A few examples

**DOI:** 10.4103/0019-5545.42404

**Published:** 2008

**Authors:** C. Shamasundar

**Affiliations:** 250, 43rd Cross, 9th Main, 5th Block, Jayanagar, Bangalore - 560 041, Karnataka, India

**Keywords:** Mental health, mind, Indian mythology, miseries and sufferings, consciousness

## Abstract

The ancient Indian concepts and paradigms relating to mental health are holistic and cover aspects that have been neglected by the modern mental health literature. The latter can borrow, study, and incorporate them in their text books to advantage. The current trend in mental health research is heavily biased in favour of biological aspects of psychological phenomena neglecting the basic entity, the mind. Correction of this partisan tilt is urgently needed.

Ancient concepts prevalent in any culture have always influenced the development of knowledge, especially in the field of psychology. Examples are to be found in the contribution of such pioneers as Freud and Jung. Moreover, in the last few decades, there has been a large-scale borrowing of eastern concepts in the practice of mental health. This fact was the basis for the author to explore some of the ancient Indian literature.

The subject matter in this write-up is based on the material that I collected during my sabbatical study in 1987-1988 funded by NIMHANS, Bangalore. The topic of that study was “Mental Health Concepts in Indian Mythology.” The ancient Indian scriptures studied were: the two Epics of Ramayana and Mahabharatha, principle Puranas, major Upanishads, Jataka stories, Jaina stories, and Ocean of stories (Bruhath-Katha-Saritsagara).

The mental health concepts collected during the study contributed to a change in my professional “assumptive-world.”Earlier, I was pained by the glaring absence of such commonly useful concepts as “mind,” “will-power,” etc. in our standard psychiatric textbooks. After the study, I was wonder struck at the richness, wholeness and intense practicability of paradigms and wisdom contained in ancient Indian literature, and its potential to enrich our professional knowledge.

The topic of Indian concepts relating to mental health is very vast. Hence, I will limit my presentation to a few examples to serve as samples of the wide variety of material that is in store for serious students who wish to explore.

The examples in this presentation are inter-related, and an integral whole may emerge from this presentation. I hope that the readers will become interested in this area of knowledge.

In this write-up, I have deliberately refrained from ritualistically quoting references for those facts which are common knowledge among mental health professionals.

## Mind, an object of academic apartheid

Our current professional literature seems to be subject to a kind of academic apartheid in the form of avoiding usage and coverage of such common and useful concepts as mind, “will power,” etc. Though avoiding the use of the word “mind,” the textbooks describe two attributes of mind, namely, Kurt Lewin's concept of field-like property, and Freud's concepts of consciousness, unconsciousness, and libidinal energy.

In contrast to our textbooks, Encyclopedia Britannica is more generous in using the word “mind ” and describing more of its attributes: (i) immaterial nature, (ii) related to personal identity and subjective experience, and (iii) having introspective ability.

Ayurveda and Indian scriptures accord mind the highest tribute, next only to God in terms of its immense potentialities. Apart from the attributes of conscious and creative energy, mind is also accorded an immaterial nature, by virtue of its association with soul in a physical body.

## Attributes of mind

From the above different sources, the attributes of the mind are: (i) conscious-unconsciousness, (ii) self-identity, (iii) field-like properties, (iv) energy or power, and (v) immaterial nature by virtue of its association with soul in physical body. For our purpose, these can be considered as tentative hypotheses. Each of these has corroborative evidences in the modern science literature. Few examples of such evidences are given below.

- Consciousness-unconsciousness
  - The projective tests used in clinical and experimental psychology are based on the dynamics of unconscious processes influencing the subject's responses to ambiguous stimuli.
  - The subconscious phenomenon of subliminal stimulation is well known for many decades.
  - The widely accepted phenomena of defense mechanisms are processes of the unconscious.
- Self-identity
  - Most of human behavior is influenced by “self-concept” or “self-image” of which the individual is generally unaware.
  - In a study reported by Sir Eccles, the motor cortical neurons of a conscious individual were stimulated resulting in a limb movement. The subject acknowledged the movement but, disowned it saying, “I did not move the limb.…”
- Field-like and energy (or power)-like properties of the mind
  As said earlier, Ayurvedic and traditional Indian concept of mind accord it an almost limitless omnipresent, field-like quality, besides omnipotent, and omniscient qualities. The reason why a common man does not experience these qualities according to the scriptures, is due to the mind being overwhelmed by preoccupation with materiality and attendant loss of required subtleness and sensitivity. Following are a few, selected evidences in modern scientific literature to indicate these qualities:
  - *Mind-mind interactions*: An experiment reported by Krippner and Ullman[1] involved the dream content of one individual being influenced by the mental imagery in another who is spatially isolated. In an experiment reported by Rebman and Radin,[2] the state of arousal in the staree (the subject being stared at) as monitored by measurable parameters correlated with the staring by the starer (subject who stared at the staree at designated intervals). Also, the phenomenon of “personal space” is extensively studied as reviewed by Scott.[3]
  - *Mind-matter interactions*: It is possible to influence the generation of numbers in an electronic random event generator (REG), and it is possible to influence by thought the damping rate of a freely swinging pendulum as reported in *Journal of Scientific Exploration*.[4,5]
  - The effect of expectation (also known as expectancy-effect, placebo-effect, pygmallion-effect, motivation, or positive-thinking, etc.) is well known to medical and mental health professionals. What an individual expects to happen will happen more often than can be accounted for by chance, depending upon the strength and intensity of one's expectation. In the field of sports, it is known as “will-power.” Here the strength and intensity represents the absence of doubts or other negative connotations to expectation.
  - These types of investigations are strengthening the ancient Indian concept of “mind-over-matter”.
- Mind's association with soul in the physical body
  This issue borders on the metaphysical dimension and is thus subject to controversy. Yet, there is extensive corroborative data to support this thesis in the form of pre-birth memories and reincarnation type cases. Since the last few decades, a large number of corroborating investigators have laboriously collected data of nearly 3000 cases from all over the world. After thorough investigations of each and every case, their conclusion is: “transmigration of the soul is a viable explanation for the phenomenon studied.” These studies give credibility to the ancient Indian concept of mind's association with soul in a physical body. Such was the impact of these studies pioneered by Late Dr. Ian Stevenson that an entire issue of the Journal of Scientific Exploration [Spring 2008, 22(1)] is devoted to description of his research by different authors.

## Mind-body relationship

The World Health Organization (WHO) definition of mental health (as “.…physical, mental and social well-being,…”) integrates physical and mental health into a holistic scheme. But, this integration in definition has not yet percolated sufficiently into teaching and clinical practice.

In contrast, the Ayurvedic texts, in their first few chapters, describe creation according to ancient Indian ideas. According to these ideas: (i) The mind with its creative potential was the first entity that came into being, and every thing else was created subsequently. (ii) The physical body is just a grosser, material replica, or “image” of the mind. This is the basis of one of the classical Indian doctrines of “mind-over-matter.” (iii) Mind and body are on an immaterial-material continuum, and mutually influence each other.

The close association of mind and body according to Ayurveda is evident from following facts from Ayurvedic texts. It has to be noted here that modern concepts of psyche and soma are in Ayurveda too in the form of “gross-physical-body,” and the “subtle-mind.” But, the difference between them is believed to be only in the degree of “grossness-sublity,” hinting at a continuum. Thus, the present day concept of psychosomatic medicine is implied in Ayurvedic teachings according to which:

- Every disease has a number of etiologic factors.
- Every physical disease has one or more psychologic factors as etiologic agents.
- The psychologic-etiologic factors are: fear, anger, grief, greed, pride, jealousy, etc. It is interesting that greed and pride are considered as etiologic factors for illnesses. The implication according to this thesis is that none of us are free from susceptibility to illnesses.
- Certain qualities of living relating to values, attitudes and behavior promote health. They are: courage, righteous living, and ability to control own behavior to ensure righteous living. According to Indian scriptures, “righteousness” is a set of attitudes and behavior that promote maximum benefit in the long run to all life. What is righteous in any given situation depends on time, place, persons involved, and context (circumstances), and can be difficult to decide for common people. But, this wisdom of righteousness can be acquired by spiritual practice. However, for common people, adherence of moral code of conduct ensures least deviance from righteousness.

It is interesting to note that the components of righteous living are opposites of illness promoting qualities mentioned above as fear, greed, jealousy, anger, grief, pride, etc. The implications of Ayurvedic points of view are:

- Psychologic factors play etiologic role for every physical illness.
- Thus, all medicine inevitably becomes “psychologic medicine,” or psychosomatic medicine.
- An individual's practice of social-moral values has positive role on one's state of health.
- Consequently, an individual is responsible for one's state of health or illness. Thus, there are no genes, chemicals, or other external agencies which can be blamed for one's illness.

### Evidences for mind-body relationship

There are sufficient evidences in modern health literature which corroborate the above principles of mind-body relationship. Few examples of these evidences are:

- Correlations of onset or exacerbations of illnesses with: (i) Adverse life events. (ii) Distressing mood-states. These type of correlations apply equally well to motor vehicle accidents also.
- Correlations of bio-chemical as well as immune-response functioning, with psychologic states.
- Correlations of course and outcome of illnesses with such psychologic factors as hope, motivation, confidence (in the therapist), etc. These correlations have come to be recognized as “placebo-effect,” “expectance-effect,” or even as “will-power,” even though medical literature has yet to acknowledge the last concept. Most striking ones among them show the psychologic differences between cancer-survivors and nonsurvivors as reviewed by Manos and Chriskis.[5]

Ever increasing number of evidences of such nature are being reported covering an ever wider range of illnesses. Hence, we can confidently repeat what was said earlier: “all medicine is psycho-somatic medicine.” An extension of this thesis is that physical health is a sequel or result of and dependent on mental health.

## Mental health

Different descriptive references to mental health in textbooks converge on to four following parameters:

1. *Appropriateness of emotional responses*. Appropriateness is in reference to prevailing context or circumstances. Of course, the “prevailing context” may also have an historical back ground.
2. *Adjustment with self and others*. (a) Adjustment with self refers to a state of harmony among all components of one's psychologic apparatus. Examples of these components are values, attitudes, beliefs, feeling, thinking, and action. This requirement is similar, if not the same as the Indian concept of “manasa-kaya-vaacha,” which in turn is similar to the meaning of genuinity. (b) Adjustment with others pertain to a state of interactional harmony with other members of the family, social milieu, etc.
3. *Functional integrity*. This has two dimensions. The first is cross-sectional integrity, ensuring reliability in all areas of one's functioning. The second is the temporal or longitudinal consistency over time under differing circumstances.
4. Self-actualization as described by Abraham Maslow, which means achieving one's full potential. A corollary of this component is: any under-achievement in respect of one's potentialities constitutes a negative value to mental health. In other words, *state of mental health has to be achieved*, and is not a free gift to a passive individual.

### Definition of mental health:

According to ancient Indian scriptures, an ideal person is expected to manage one's life in spite of adversities of any nature. The following integrated definition of mental health is based on the descriptions from mental health textbooks and from the description of an ideal person from the Indian scriptures.

A mentally healthy person attends to one's legitimate duties in personal, family, social and occupational areas fulfilling spiritual, affectional, and material needs of self and family in harmony among one's role functions, one's abilities and limitations, prevailing circumstances and righteous means with sincerity and honesty, hope and confidence, and contentment.

The above description of a healthy individual is holistic in its coverage and at the same time, operational. Also, for those of us who have some how managed to lead a relatively comfortable life by some means or the other, this state of mental health seems frighteningly tough.

### Corollaries to definition of mental health:

The following are a few corollaries to the integrated definition of mental health:

1.
  - Mental health is not a passive state. It can never be taken for granted. An individual has to actively achieve a state of mental health. But, the modern concept of “self-actualization” as a component of mental health lends some support to this difficult task. This idea is a bitter pill, especially in the present day industry and commerce dominated “easy-culture.”
  - If some one has remained asymptomatic without having been challenged by difficulties or illnesses, there is no guarantee of subsequent mental health.
  - Therefore, assessment of mental health must be based on the stresses an individual has faced and how he/she has coped with them.
  - This requires re-orientation and re-organization of methods of assessing the state of mental health.
2.
  - Mental health is an evolving, dynamic process that has to be actively achieved in “living-learning” situation of life, just like we learn our medicine by “bed-side” clinical work.
  - There is always a “price to pay for mental health”: difficulties to face, disappointments, and setbacks to brave, certain distresses to bear with dignity, and keep continuously refining one's coping skills.
  - Only those individuals who have been challenged by adversities in life and have learnt the coping skills have the potential to remain mentally healthy within the limits of their abilities.
  - Thus, the state of an individual's mental health has two dimensions to it: (i) The longitudinal, which is the individual's potential to be mentally healthy, which is dependent upon the person's psychologic apparatus as well as the coping skills that have been learnt. (ii) The cross-sectional, which is dependent upon the severity of stress in relation to one's potential.
  - Interestingly, there is a proverb in Kannada language which may have similar counter parts in other languages. Its translated meaning is “test a friend in difficulties.” It means truth about a friend's friendship becomes evident only in moments of one's difficulties. In the same way, an individual's state of mental health potential must necessarily be assessed in respect of how he/she has coped with difficult situations, when “things have went wrong.”

**Concept of contentment** is much misunderstood. This needs some explanation. It constitutes one of the basics of ancient Indian ideal. It is not complacency, nor a passive acceptance of a given state of affairs, nor is it a state of stagnated misery. It is an intensely dynamic acceptance of results of one's efforts in moment-to-moment struggle of life:

- Every individual has a legitimate right to aspire to shape one's future toward betterment in all aspects of life and strive toward a legitimate goal. He/she is also duty bound to do so. This requirement is contained in the integrated definition of mental health described above.
- But, at the same time, one must be ready to accept any failure or disappointment with composure, dignity and gracefulness without resorting to pathologic responses like blaming oneself or others, regretting, or brooding on the past, etc.
- When an individual encounters failure, he/she should review: (i) the lessons to learn from the failure; (ii) one's goals and objectives; (iii) one's abilities and limitations; (iv) means and methods, and (v) future course of action.

It is thus that contentment is a dynamic process based on an attitude of cross sectionally respecting and accepting a result at any given time.

## A dilemma relating to mental health

### Inevitability of miseries and sufferings:

There is a tendency among people, including mental health professionals to take the state of mental health for granted. But, there is a dilemma in the form of an assertion by Indian Philosophy: in human life, miseries and illnesses are inevitable.[6] This thesis is a logical consequence of *Samskara-theory*: a soul takes birth only for the purpose of undergoing the fruition of its accumulated lot of *Samkaras* (impressions of actions). There is no such thing as exclusive happiness or sorrow; there never is one without the other, even though people pretend as if the contrary is true. In this context, it is interesting to note that the meaning of the Sanskrit word Ayurveda (ancient Indian system of medicine) is: science of longevity. Ayurveda is not a science of disease free life.

The fact of inevitability of miseries and sufferings has three interesting aspects. (i) First, every individual's life is a living proof of this fact. (ii) Second, people cherish a delusional ideal to lead a symptom-free and difficult-free life, and even demand it as their birthright whenever it suits them to do so. The pharmaceutical and other commercial enterprises keep continuously brainwashing the population to cherish this delusion as an ideal. (iii) Third, the fact of inevitability of miseries and difficulties in life is not adequately acknowledged by the mental health profession, even though it has recently begun to acknowledge “daily hassles.”

If miseries and illnesses are inevitable in human life, what then is mental health? The only logical way of effectively solving this conceptual dilemma is to accept that:

- State of mental health should not be dependent on the presence or absence of illnesses or difficulties, and should transcend both. Thus, it should be possible for an individual to be mentally healthy in spite of difficulties and/or illnesses.
- It should also involve sincere management of illnesses and/or difficulties to the best of one's abilities and circumstances. In such an attempt, the issue of success or failure has already been dealt with while explaining about contentment.
- Thus, state of mental health is a set of attitudes and coping skills.

## A dilemma relating to spiritual practices and mental health

Recently, both within the mental health profession and otherwise, there has been an ever increasing adoption of practices relating to spiritual teachings for purposes ranging from management of stress to curing one's illnesses. The practices range from *yogasanas, pranayama*, meditation, or combinations of these under different proprietary names. Extensive investigations have been done about such practices and the results support beneficial effects in favor of a sense of wellbeing.

But, in the ancient Indian scriptures, there are repeated admonitions about spiritual practices for material, wordly purposes:

- Mere avoidance of miseries and the like is not the goal of human life. The inevitability of miseries and illnesses is already mentioned above. According to ancient Indian teachings, they are the means to salvation. Sage Narada instructs King Prithu[7,8] that human welfare does not consist of destruction of miseries and attainment of worldly happiness.
- They are meant only for spiritual purposes like “god-realization.” Purity of objective or aim without adulterating the objective with other motives is essential in such pursuits. In some context, sage Manu explains to sage Bruhaspati[9] that desiring something other than the highest aim of human life is a serious obstruction to the path of spirituality.
- They should not be used for worldly purposes like avoiding or overcoming illnesses or mitigating miseries related to affairs of living. Examples of such admonitions are by Lord Shiva in *Shiva Purana*[10] and Sage Vashishtha in *Yoga Vashishtha.*[11] In one instance, Lord Shiva even punishes a devotee for misusing spiritual practice for worldly gains.[12]

The recurrent message is: (i) spiritual practice is meant for the highest objective of human life beyond material existence; (ii) at the worldly level, miseries and illnesses can and should be dealt with by numerous material means available, and (iii) a state of serenity, etc. is a natural by-product of spiritual practice. But, that state in itself should not be the objective. That is, the sole purpose of so-called “peace of mind,” or similar purposes should not become objectives of spiritual practice. This is the paradox.

## How to achieve mental health without spiritual practice?

I have shown in an earlier paper[13] that components of ideal human behavior are similar to those of desirable therapist qualities, which in turn are similar to mental health promoting qualities.

Therefore, a state of mental health can be achieved by practicing and inculcating ideal human qualities which I believe to be similar in all cultures.

## Paradox of consciousness-related studies

Extensive studies on consciousness and related phenomena are being conducted using sophisticated instruments. Examples of the phenomena that are being attempted to study are levels of consciousness, states of meditation, and various psychologic tasks. Examples of complex instruments are multichannel EEG recorders, magnetic resonance imaging (MRI), and positron emission tomography (PET) scans with respective software. The physical instruments can however record physical correlates of the activities or states of consciousness. But, can the physical instruments ever record the “immaterial” activities of the immaterial mind? This is the paradox.

Hence, to study mind and its various modalities and activities, it is essential to do the following:

- Legitimize subjective experiences as tools of scientific enquiries, and develop methods of recording and analyzing subjective experiences in a reliable manner.
- Develop methods of introspection as a legitimate method of studying psychologic phenomena.

Of course, many modern mental health professionals have already advocated these measures which are yet to acquire widespread usage in practice.

## Urgent future needs

- Review the available knowledge on mind and related phenomena from religious, spiritual, and other sources (which can be called “peripheral sources”) and incorporate the same in standard textbooks, even if as alternate opinions. This will offer an opportunity for interested research workers to study them further instead of keeping the professionals blind to their existence.
- Encourage and create opportunities for the concepts relating to mind from peripheral sources to be subjected to critical study and research, instead of neglecting them.

I have suggested in my two earlier papers[14–16] two examples of such a need, namely, phenomena related to empathy-telepathy and love.

These needs highlight yet another paradox of “neglecting the gold mine in our own backyard,” or the paradox of misplaced emphasis.

## Paradox of misplaced emphasis

In the 1930s and 1940s of last century, physicists involved with quantum theory had difficulty in explaining certain bizarre consequences of that theory. So, they used the concept of consciousness in a sense beyond what we mental health professionals ascribe to that term. By consciousness, we mean just awareness. By their ‘Copenhagen Interpretations', quantum physicists had implied a quality of intention to it!

As far back as in 1974, a term “para-physics,” similar to the term “para-psychology” was coined by scientists of natural sciences to study phenomena that are not studied by conventional sciences and methods.[17]

In 1979, a program called “Princeton Engineering Anomalous Research (PEAR)” was started in Princeton University to study anomalous phenomena including such phenomena as mind-mind and mind-matter interactions.[17] Even biologists like Rupert Sheldrake[18] are researching remarkable anomalous mental abilities in animals that are difficult to explain.

The paradox is: while natural scientists are busy since few decades studying hitherto neglected phenomena relating to consciousness, we the mental health professionals are still busy studying the chemistry of the nervous system, ignoring such concepts as mind and its “immaterial” manifestations.

## THE HOPE

It is earnestly hoped that the metal health profession will soon reorient its emphasis about expanding its knowledge base and areas for research by seriously studying ancient Indian concepts relevant to mind and mental health and incorporating them into its body of knowledge.

## References

[1] Krippner S, Ullman M (1970). Telepathy and dreams: A controlled experiment with EEG and EOG monitoring. J Mental Nerv Dis.

[2] Rebman J, Radin D (1995). Parapsychological association convention. J Sci Expl.

[3] Scott AL (1993). A beginning theory of personal space boundaries. Perspect Psychiatr Care.

[4] Dunne BJ (1994). Series position effects in random event generator experiments. J Sci Expl.

[5] Nelson RD (1994). A linear pendulum experiment: Effects of operator intention on damping rate. J Sci Expl.

[6] Manos M, Christkis J (1985). Coping with cancer: Psychological dimensions. Acta Psychiatr Scand.

[7] Mitra VL (1981). Yoga Vashistha, Book 6, chapt 83.

[8] Tagare GV, Shastri JL, Skanda (1979). The bhagavatha purana.

[9] Ganguli KM (1970). The Mahabharata, Shanthi Parva, chapt. 206.

[10] Shastri JL (1983). Shiva Purana, Section II, chapts. 10-11.

[11] Mitra VL (1891). Yoga Vashistha, Book 6, chapts. 41-43.

[12] Penzer NM (1968). Ocean of stories.

[13] Shamasundar C, Dwivedi K (1997). Psychotherapeutic paradigms from Indian mythology. Therapeutic use of stories.

[14] Shamasundar C (1999). Understanding empathy and related phenomena. Am J Psychother.

[15] Shamasundar C (2001). Love, praxis and desirable therapist qualities. Am J Psychother.

[16] Berger LS (2001). Commentary to praxis paper. Am J Psychother.

[17] Beichler JE (2001). To be or not to be! A paraphysics for the new millennium. J Sci Expl.

[18] Sheldrake R (1995). Seven experiments that could change the world, Fourth estate.

